# *Enterococcus faecalis* shifts macrophage polarization toward M1-like phenotype with an altered cytokine profile

**DOI:** 10.1080/20002297.2020.1868152

**Published:** 2021-01-04

**Authors:** Mohamed Mohamed Elashiry, Fucong Tian, Mahmoud Elashiry, Rana Zeitoun, Ranya Elsayed, Matthew L. Andrews, Brian E. Bergeon, Christopher Cutler, Franklin Tay

**Affiliations:** a Department of Periodontics, The Dental College of Georgia, Augusta University, Augusta, GA, USA; b Department of Endodontics, Faculty of Dentistry, Ain Shams University, Cairo, Egypt; c Department of Endodontics, The Dental College of Georgia, Augusta University, Augusta, GA, USA; d Department of Oral Biology and Diagnostic Science, The Dental College of Georgia, Augusta University, Augusta, GA, USA; e Department of Fixed Prosthodontics, Faculty of Dentistry, Ain Shams University, Cairo, Egypt

**Keywords:** Apoptosis, *Enterococcus faecalis*, infection, intracellular, macrophage, plasticity

## Abstract

**Background**: The macrophage is an innate immune defense cell involved in pathogen recognition and clearance.

**Aim**: In view of the diversity of the macrophage phenotype and function, the present study investigated how *Enterococcus faecalis* infection affects the differentiation, phenotype and cytokine profile of macrophages.

**Methods**: Murine bone marrow-derived stem cells were co-cultured with *E. faecalis* before and after differentiation. Macrophage M0 polarization towards M1 or M2 was initiated at day 6 by addition of LPS and INF-γ, or IL-4 and IL-13, respectively.

**Results**: *E. faecalis* did not inhibit macrophage differentiation and were identified within macrophages. Viability of the macrophages infected with *E. faecalis* prior to differentiation was enhanced, evidenced by apoptosis inhibition, as was expression of CD38 and IRF5 proteins, indicators of M1-like polarization. These M1-like macrophages expressed an aberrant cytokine mRNA profile, with reduction in inflammatory cytokines IL-1β and IL-12 and increase in regulatory cytokine IL-10. No changes in TNF-α or TGF-β1 were detected, compared with the control groups. This atypical M1-like phenotype was retained even upon stimulation with growth factors that normally trigger their development into M2 macrophages.

**Conclusions**: These findings suggested that *E. faecalis* infection of bone marrow-derived stem cells during differentiation into macrophages induces an atypical M1-like phenotype associated with intracellular bacterial survival.

## Introduction

*Enterococcus faecalis* are facultative anaerobic Gram-positive cocci that colonize the human gastrointestinal tract. These bacteria do not cause deleterious effects in healthy individuals. However, by disrupting the host immune response, *E. faecalis* can enter the bloodstream and spread to different body parts. In immunocompromised individuals, this may result in life-threatening diseases such as meningitis, endocarditis, serious wound infection or urinary tract infection [1]. Because of their virulence factors and ability to form biofilms, *E. faecalis* can survive in harsh conditions such as hypoxia and resist eradication by antimicrobial agents, ultraviolet light irradiation, acids, alkalis or high temperature [2,3]. *Enterococcus faecalis* utilize cytolysin (hemolysin/bacteriocin), aggregation substance, surface adhesins, gelatinase or protease, sex pheromones and lipoteichoic acid for survival, interacting with other bacteria and modulating the immune response of their host [4,5].

From a dental perspective, *E. faecalis* is attributed to the failure of root canal-treated teeth and the emergence of post-treatment secondary infections [6]. *E. faecalis* has been identified in 4 to 40% in cases with primary endodontic infections, a higher frequency was reported in cases with unhealed periapical lesions. Other studies showed the prevalence of *E. faecalis* in endodontically treated teeth with periapical infections to range from 24 to 77% [7]. Some reports showed that *E. faecalis* was the only microorganism found in the root filled teeth with periapical infections [8]. The persistence of periapical infections after root canal treatment and how these bacteria overcomes the host immune response are still not widely explored.

The effect of *E. faecalis* on macrophages and their modulation of the immune system have been a topic of interest over the last few years [9]. Macrophages are one of the frontline defense cells of the human immune system. They are critical to both acute and resolving immune responses. Upon infection, circulating monocytes are recruited to the site of infection where they differentiate into macrophages and/or dendritic cells. Macrophages are activated into a pro-inflammatory M1 phenotype upon recognition of pathogen-associated molecular patterns derived from the bacteria, via Toll-like receptors present on their surfaces. Macrophages with this phenotype secrete inflammatory cytokines that aid in the phagocytosis of pathogens and activation of T cells [10]. Prior to the discovery of the phenotypic heterogeneity of macrophages, it was believed that macrophages have only a pro-inflammatory role in innate immunological defense [11]. With the advent of the macrophage polarization concept, it is now known that macrophages have a dual role in inflammation. These cells exhibit remarkable plasticity, which enables them to adapt to different functional programs in response to the nature of the cytokines present at the infection microenvironment. In the presence of INF-γ secreted by T helper-type 1 (Th-1) cells and/or lipopolysaccharides (LPSs) derived from the cell wall of Gram-negative bacteria, macrophages differentiate into the M1 pro-inflammatory phenotype that is destined for pathogen recognition and phagocytosis. In the presence of interleukin-4 (IL-4) and/or IL-13 secreted by T helper-type 2 (Th-2) cells, macrophages are polarized into the M2 anti-inflammatory phenotype. The M2 macrophages have different subtypes, most of which are involved in wound healing and resolution of inflammation [12]. It is now believed that a continuum of tissue macrophage polarization states exists. These cells are overlapping with each other in terms of gene expression and function in response to a plethora of environmental stimuli [13].

*Enterococcus faecalis* can survive within macrophages for long periods after infection [14] because of their resistance to phagosome acidification and autophagy inhibition [15]. They have been reported to inhibit apoptotic cell death of macrophages to facilitate their continuous survival [16]. These bacteria also delay wound healing after infection. This may be attributed to suppression of the host immune response, increase in bacterial titer and colonization of the wound surfaces [9,10].

To date, the effect of *E. faecalis* on macrophage polarization during their differentiation is not entirely understood. Accordingly, the objective of the present study was to investigate the effect of prolonged infection of macrophage precursors with *E. faecalis* on macrophage polarization. The rationale for infecting macrophages for longer than 24 h was to emulate the clinical scenario of chronic infection. The cytokine profiles secreted by the differentiated macrophages were also examined to see if they were typical of M1 and M2 lineages. The null hypothesis tested was that prolonged *E. faecalis* infection of macrophage precursors has no effect on the phenotype of the differentiated macrophages and their cytokine expression profile.

## Materials and methods

### Murine bone marrow-derived stem cells and macrophage differentiation

Stem cell retrieval from mice and other procedures performed in the study were approved by the Institutional Animal Care and Use Committee at Augusta University (protocol #2013-0586). At day 0, the tibia and fibula of 9–11-week-old C57BL/6 mice (Jackson Laboratory, Bar Harbor, ME, USA) were extracted after euthanasia of the animals by CO_2_ asphyxiation. Cells were collected from the bone marrow by inserting the tip of a short-length 27-gauge needle into the bone ends. The retrieved cells were cultured in Rosewell Park Memorial Institute (RPMI)-1640 medium supplemented with 10% fetal bovine serum, 1% penicillin/streptomycin and 100 μL/mL β-mercaptoethanol (complete medium; Gilbo, Thermo Fisher Scientific, Waltham, MA, USA). Red blood cells were removed with ACK lysis buffer (Invitrogen, Thermo Fisher Scientific). The remaining cells were washed twice, counted and placed into tissue culture dishes. Each dish received a density of 1 × 10^6^ cells/mL of complete medium. Macrophage colony-stimulating factor (M-CSF; Miltenyi Biotec Inc., Auburn, CA, USA; 10 ng/mL) was added to induce the differentiation of bone marrow stem cells (BMSCs) into macrophages. The cells were incubated at 37°C and 5% CO_2_ for 8 days, with replenishment of the complete medium and M-CSF every three days [17]. Floating cells were discarded during medium change, leaving cells that adhered well to the culture dishes.

Macrophage polarization was initiated at day 6 by supplementing the replenished medium with growth factors according to the type of macrophage to be generated. For naïve macrophages (M0), only M-CSF was added to the medium. For M1 activation from M0, 100 ng/mL of LPS (MilliporeSigma, MO, USA) and 50 ng/mL INF-γ (Miltenyi Biotec) were added to the medium. For M2 activation from M0, 10 ng/ml of IL-4 and 10 ng/ml of IL-13 (Miltenyi Biotec) were added to the medium. The activation factors were added 24 h prior to analysis [17].

### Bacterial preparation and infection of BMSCs

*Enterococcus faecalis* (ATCC 29,212; American Type Culture Collection, Manassas, VA, USA) were cultured in sterile brain heart infusion broth (Bacto™ Brain Heart Infusion, Becton, Dickinson and Company, Franklin Lakes, NJ, USA) and kept in an aerobic chamber for 24 h at 37°C and 5% CO_2_. Infection of BMSCs during differentiating into macrophages (pre-M0 + *E. faecalis*) was performed at day 0 with multiplicity of infection (MOI; ratio of bacteria to infection target) of 1 via adjusting the bacteria density to 10^8^ cells/mL using an ultraviolet/visible light spectrophotometer (BioMate™ 3S, Thermo Fisher Scientific) at an optical density of 1 at 600 nm. Infection was maintained throughout the study by adding an additional dose of *E. faecalis* at day 6 with the same MOI. The rationale of using an MOI of 1 was to mimic chronic infection for a prolonged time with a low quantity of bacteria; a similar MOI was used for examining *E. faecalis* infection of BMSC-derived dendritic cells [18]. Infection of the already differentiated macrophages (post-M0 + *E. faecalis*) was performed at day 6 with an MOI of 1, to correlate and compare the effect of *E. faecalis* on macrophages before and after their differentiation. The effects of the M1 and M2 environment on the pre-M0 + *E. faecalis* were analyzed at day 6. Twenty-four hours prior to analysis, INF-γ and LPS were added to the medium for M1 activation. Likewise, IL-4 and IL-13 were added for M2 activation.

### Ultrastructure of E. faecalis uptake by differentiating macrophages

At day 7, pre-M0 + *E. faecalis* at day 0 were collected by dissociating the adherent cells from the culture dishes and centrifuged. The cell pellet was fixed with 4% paraformaldehyde and 2% glutaraldehyde in 0.1 M sodium cacodylate buffer (pH 7.4), post-fixed with 2% osmium tetroxide, stained *en bloc* with 2% uranyl acetate, dehydrated with a graded ethanol series and embedded in epon-araldite resin. Seventy nanometer thick sections were prepared, collected on copper grids and stained with 2% uranyl acetate and Reynold’s lead citrate. The stained sections were examined by transmission electron microscopy (TEM; JEM 1230, JEOL USA Inc., Peabody, MA, USA) at 110 kV and imaged with an UltraScan 4000 CCD camera (Gatan Inc., Pleasanton, CA, USA).

### Bacteria uptake by differentiated macrophages

After 8 days of macrophage culture in complete medium with differentiating factors, M0 macrophages were infected with *E. faecalis*, labeled with 10 μM carboxyfluorescein succinimidyl ester [CFSE; eBioscience, Thermo Fisher Scientific]) as reported [19], at an MOI of 1 for 6 h. The CFSE-*E. faecalis* were added to the M0 macrophages at a cell density of 5 × 10^5^ and kept in the aerobic chamber at 37°C and 5% CO_2_ atmosphere for 6 h. The macrophages were visualized with a bright field in the video mode to monitor bacteria uptake by endocytosis, using a Zeiss LSM 780 inverted confocal microscope (Carl Zeiss AG, Oberkochen, Germany).

### Flow cytometry

The macrophages were collected at day 7 by applying Cell Dissociation Buffer Enzyme-Free, PBS (Gibco) to the adherent cells. The dissociated cells were centrifuged and the cell pellet was re-suspended twice in Flow Cytometry Staining Buffer (Invitrogen). The macrophages were then incubated with the corresponding fluorophore-conjugated antibody (see next paragraph) at the manufacturer’s recommended concentration for 30 min each. Data were acquired using the Analyzer Flow Cytometer (MACSQuant®, Miltenyi Biotec) and analyzed using the MACSQuantify software (Miltenyi Biotec).

The CD11b antibody (anti-mouse, VioBlue, monoclonal; REA592) and the F4/80 antibody (anti-mouse, allophycocyanin (APC), monoclonal; REA126) were used as markers for macrophage differentiation. The CD38 antibody (anti-mouse, fluorescein isothiocyanate (FITC), monoclonal; REA616) was used as the marker for M1 polarization. The EGR2 antibody (anti-mouse, phycoerythrin (PE), monoclonal; REA869) was used as the marker for M2 polarization [20]. All flow cytometry antibodies were acquired from Miltenyi Biotec (REAfinity™ recombinant antibodies).

The effect of *E. faecalis* on macrophage apoptosis was examined using Annexin V Apoptosis Detection Set PE-Cyanine7 (eBioscience). The macrophages were suspended in 1 x binding buffer and treated with fluorochrome-conjugated Annexin V. The cells were incubated in the dark for 15 min at ambient temperature. Acquired data were analyzed using a flow cytometer and its accompanying software (Miltenyi Biotec).

### Real time-polymerase chain reaction

The RNeasy mini kit (Qiagen, Hilden, Germany) was used to isolate total ribonucleic acid (RNA) from the macrophages according to the manufacturer’s instructions. Briefly, the cell pellet was disrupted by adding an appropriate volume of Buffer RLT. Ethanol was added to the lysate and mixed well. The sample was then transferred to an RNeasy Mini spin column placed in a 2 mL collection tube. Contaminants were washed away after binding of the total RNA to the spin column. High-quality RNA was then eluted using 30 μL of RNAse-free water. The purity and concentration of the isolated RNA were evaluated using a spectrophotometer (NanoDrop 1000 UV-VIS Spectrophotometer and software Ver.3.8.1, Thermo Fisher Scientific). The RNA was reverse-transcribed to complementary deoxyribonucleic acid (cDNA) using the High-Capacity RNA-to-cDNA Master Mix kit (Thermo Fisher Scientific). The samples were then placed in Veriti^TM^ 96-Well Thermal Cycler (Thermo Fisher Scientific).

TaqMan gene expression primers specific for the IL-1β gene (Mm00434228_m1), TNF-α (Mm00443258_m1), IL-12 (Mm01288989_m1), TGF-β 1 (Mm01178820_m1) and IL-10 (Mm01299386_m1) were employed for analysis by quantitative real time-polymerase chain reaction (RT-PCR). The experiment was conducted using a StepOnePlus^TM^ RT-PCR System (Thermo Fisher Scientific). Acquired data were normalized against β-actin, analyzed using the 2^−^^ΔΔ^*^CT^* method and expressed as relative fold changes.

### Western blot

The effect of prolonged infection by *E. faecalis* on macrophage polarization was examined at the protein level using Western blot. This was achieved by analyzing the protein expression of Interferon Regulatory Factor 5 (IRF5), which increases with M1 polarization [21]. Proteins were extracted from the macrophages at day 7 by adding lysis buffer (RIPA buffer) to the cells. The cell lysate was incubated on ice for 20 min. Protein extraction was performed using 5–14% Mini-PROTEAN TGX Precast Protein Gel (Bio-Rad Laboratories, Hercules, CA, USA). The extracted proteins were transferred to polyvinylidine difluoride membranes (MilliporeSigma). The membranes were blocked with 5% non-fat dry milk in Tris-Buffered Saline-Tween (TBST) buffer and incubated overnight at 4°C with IRF5 rabbit monoclonal primary antibodies (E914Z, Cell Signaling Technology, Danvers, MA, USA) at a dilution of 1:1,000. The membranes were washed in TBST buffer and incubated with horseradish peroxidase-conjugated rabbit anti-mouse secondary antibodies for 1 h. The separated proteins were detected using enhanced chemiluminescence (Clarity Western ECL Substrate, Bio-Rad) and imaged using the ChemiDoc™ MP Imaging System (Bio-Rad).

### Statistical analyses

All experiments were repeated at least three times. Data were tested for their normality and homoscedasticity assumptions prior to the use of parametric statistical methods. One‑way analysis of variance (ANOVA) was used for multi-group comparison. Post-hoc pairwise comparisons were conducted using the Tukey test. Student T-test was used for comparison of two groups (GraphPad Prism version 7.00 for Windows, GraphPad Software, La Jolla, CA). Statistical significance was pre-set at α =.05.

## Results

### Enterococcus faecalis infects macrophages and localizes within their cytoplasm

**Figure 1.**
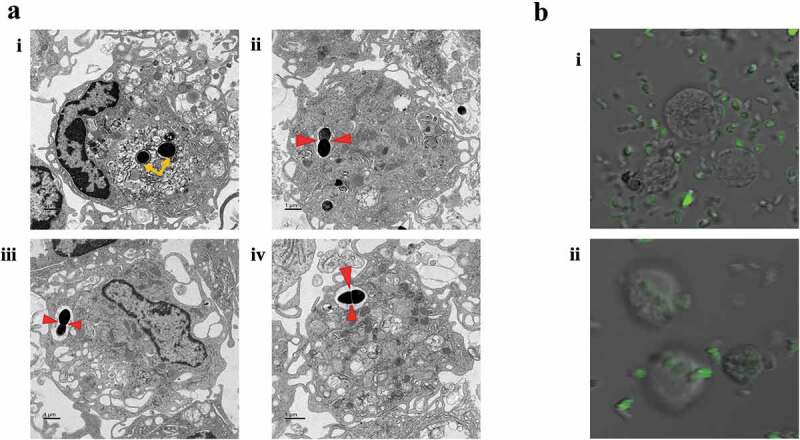
Infection of macrophages by *E. faecalis* and their intracellular localization within the cytoplasm. **A**. TEM images of *E. faecalis* infection prior to macrophage differentiation showing **i**. bacteria within the cytoplasm of a macrophage (yellow arrows), **ii–iv**. Sections with bacterial replication (binary fission; red arrows) within intracellular vacuoles. **Bi-ii**. immunofluorescent images with the uptake of CFSE-stained *E. faecalis* by fully differentiated macrophages

**Figure 2.**
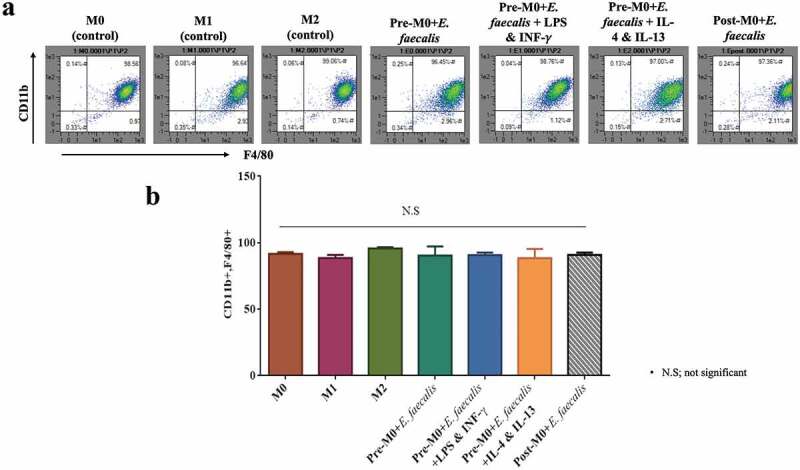
Infection of macrophages by *E. faecalis* had no adverse effect on cellular differentiation. **A**. Flow cytometry scattergrams showing the percentage expression of CD11b on the Y-axis and F4/80 on the X-axis. Cell populations in the top right corner are CD11b+F4/80+ . **B**. Bar chart of the CD11b+F4/80+ cell population in the control (M0; M1; M2) and experimental groups (pre-M0 + *E. faecalis*; pre-M0 + *E. faecalis* + LPS and INF-γ; pre-M0 + *E. faecalis* + IL-4 and IL-13; post-M0 + *E. faecalis*). Pre-M0: cells prior to differentiation into macrophages; post-M0: differentiated macrophages. Groups linked with a horizontal bar are not significantly different (*P* > 0.05; N = 3)

*Enterococcus faecalis* were identified within macrophages retrieved at day 6 (Figure 1 (Ai)). Some appeared engaged in replication (binary fission) within the cytoplasm of the macrophages (Figure 1 (Aii-iv)). Bacterial uptake by differentiated macrophages after 6 h of infection was detected by confocal laser scanning microscopy (Figure 1 (Bi,ii)). Video recording showed phagocytosis of bacteria by the macrophages (Supplementary video). *Enterococcus. faecalis* did not have any adverse effect on macrophage differentiation, evidenced by a high level of macrophage marker expression (CD11b and F4/80; 96–99.5%), with no significant difference among the seven groups (*P* = .39; Figure 2(a,b)).

### Inhibition of apoptosis in macrophages infected by E. faecalis prior to their differentiation

**Figure 3.**
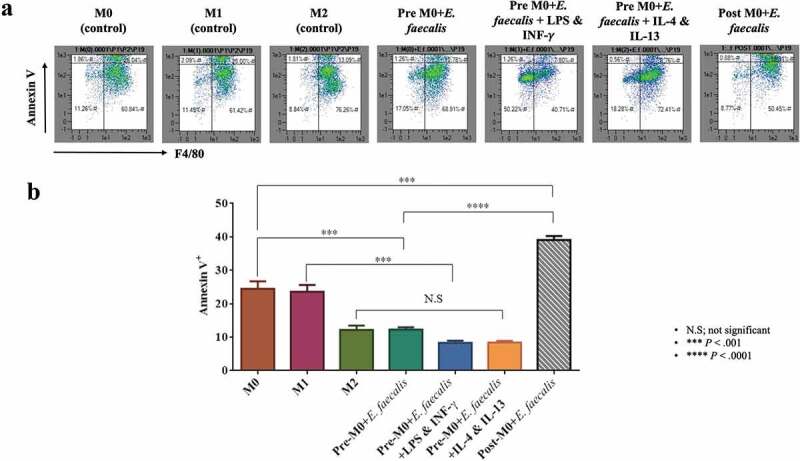
*Enterococcus faecalis* infection prior to macrophage differentiation inhibited cell apoptosis. **A**. Flow cytometry scattergrams showing the percentage expression of Annexin V (Y-axis) in the control and experimental groups. Cell populations in the top right corner are macrophages expressing the apoptosis marker **B**. Bar chart showing the mean percentage of F4/80-positive (X-axis) cell population that expresses Annexin V (N = 3)

*Enterococcus faecalis*-infected BMSCs prior to differentiation (pre-M0 + *E. faecalis*) showed a significant decrease in the expression of the apoptosis marker Annexin V at day 7. This observation was consistent in all pre-M0 + *E. faecalis* macrophage groups. However, macrophages that were infected by *E. faecalis* after their differentiation (post-M0 + *E. faecalis*) had a significantly higher percentage of a cell population that was positive to Annexin V (Figure 3(a,b); *P* < .0001).

### Enterococcus faecalis shifts macrophage polarization to the M1-like phenotype

**Figure 4.**
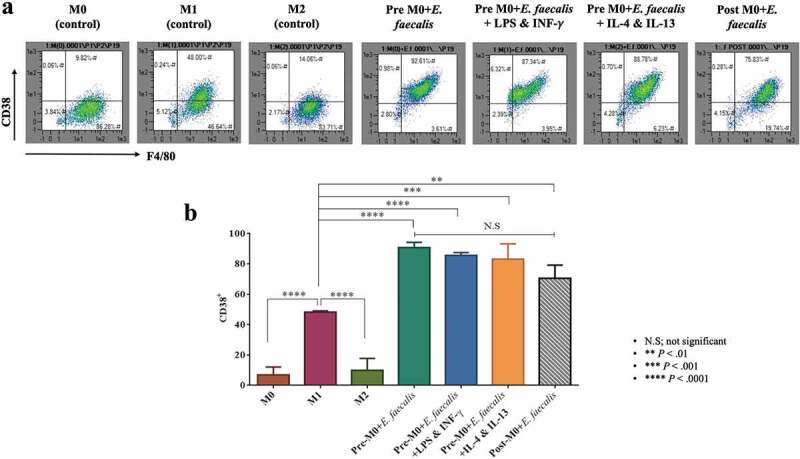
*Enterococcus faecalis* infection shifted macrophage polarization to M1-like phenotype. **A**. Flow cytometry scattergrams showing the percentage % expressions of CD38. **B**. Bar chart showing the mean percentage of F4/80-positive cell populations that expresses CD38 (N = 3)

**Figure 5.**
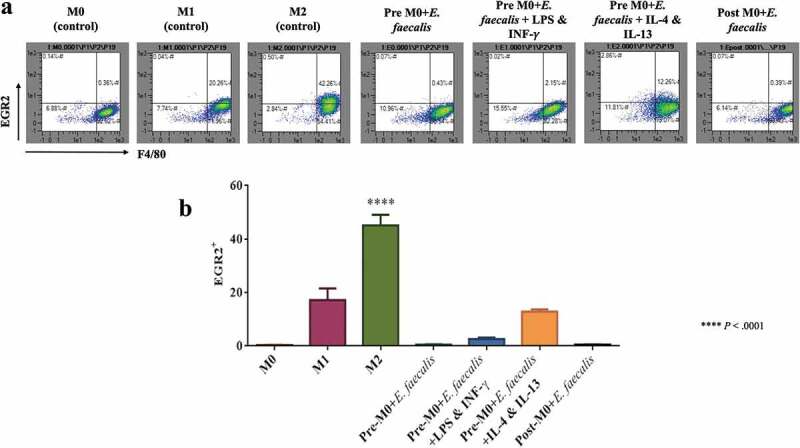
Macrophage polarization to the M1-like phenotype after infection with *E. faecalis* was evident by the lower expression of EGR2; marker for M2 macrophages. **A**. Flow cytometry scattergrams showing the percentage % expressions of EGR2. **B**. Bar chart showing the mean percentage of F4/80-positive cell populations in the control and experimental groups that are EGR2 positive (N = 3)

To detect the effect of *E. faecalis* infection on macrophage polarization, the expression of CD38; marker for M1 macrophages and EGR2; marker for M2 macrophages were examined using flow cytometry analysis [20]. Cells expressing CD38^+^ Egr2^−^ are considered macrophages of the M1-like phenotype, while cells expressing CD38^−^ Egr2^+^ are considered macrophages of the M2-like phenotype. The results showed that when macrophages were infected with *E. faecalis* before differentiation (pre-M0 + *E. faecalis*) or after differentiation (post-M0 + *E. faecalis*), the infected cells showed a shift in polarization towards the M1-like phenotype where the expression of CD38 was significantly high in all the infected groups in relation to the control groups (Figure 4(a,b); *P* < .0001), with a very low expression of Egr2 (Figure 5(a,b); *P* < .0001). These results were consistent in all the infected groups. No significant difference was detected when ‘pre-M0 + *E. faecalis*’ were exposed to the M1 environment (i.e., pre-M0 + *E. faecalis* + LPS and INF-γ) or the M2 environment (i.e., pre-M0 + *E. faecalis* + IL-4 and IL-13).

**Figure 6.**
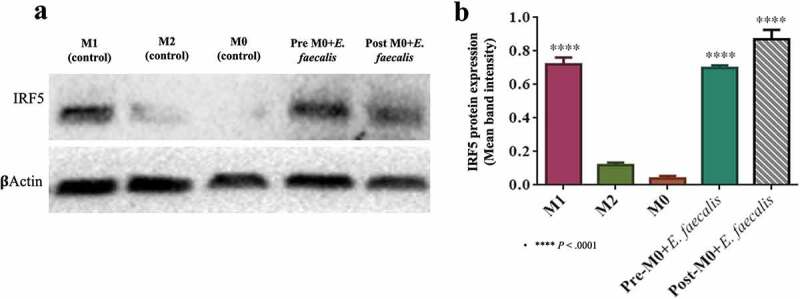
Expression of IRF5 protein in infected and control M1 macrophages. **A**. Western blot of IRF5, which is normally expressed in M1 macrophages, were expressed in pre-M0 +* E. faecalis* and post-M0 + *E. faecalis*, β-actin was used as the housekeeping protein. **B**. Bar chart showing the mean band intensity of IRF5 protein expression among the different tested groups (N = 3)

Interferon Regulatory Factor 5 (IRF5) protein expression which is highly expressed in M1 macrophages was measured via western blotting to confirm the shift of macrophage polarization toward an M1-like phenotype after *E. faecalis* infection [21]. Significantly higher expression of IRF5 was identified in the M1 control groups as well as in the infection groups (Figure 6(a,b); *P* < .0001).

### Infected macrophages showed aberrant expression of inflammatory cytokines

**Figure 7.**
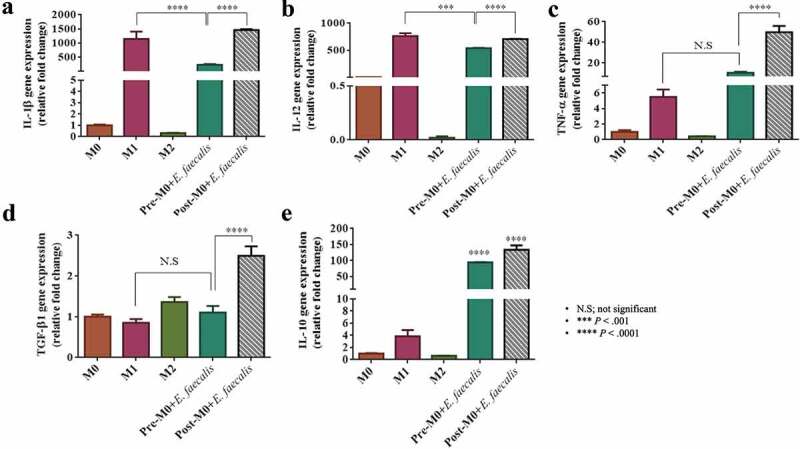
*Enterococcus faecalis* infection of differentiating macrophages induced an abnormal inflammatory cytokine profile in differentiated macrophages. Levels of mRNA expression of **A**. IL-1β, **B**. IL-12, **C**. TNF-α, **D**. TGF-β1 and **E**. IL-10. Data were normalized to β-actin (relative fold changes)

Analysis of inflammatory cytokines gene expression was performed for the M1 control groups to detect the inflammatory profile of the resultant M1-like macrophage phenotype that occurred after *E. faecalis* infection. Macrophages infected with *E. faecalis* on day 6 for 24 h (post-M0 + *E. faecalis*) exhibited a significant increase in all the inflammatory cytokines examined (*P* < .0001). However, long-term infection of macrophages prior to their differentiation with *E. faecalis* (pre-M0 + *E.faecalis*) showed an atypical inflammatory cytokine profile compared with that expressed by the control M1. This was evidenced by a significant reduction in the mRNA expression of the pro-inflammatory cytokines IL-1β and IL-12 (Figure 7(a,b); *P* < .0001). There was also a significantly higher expression of the anti-inflammatory cytokine IL-10 (Figure 7(e); *P* < .0001). No significant difference was detected in the expression of TGF-β1 and TNF-α between the infected M0 macrophages (pre-M0 + *E. faecalis*) and the control M1 macrophages (Figure 7(c,d)).

## Discussion

*Enterococcus faecalis* is a commensal bacterium residing in the gastrointestinal tract and urinary tract of the human body. This bacterium can be highly pathogenic in certain conditions, such as in immunocompromised patients [22,23]. In addition, *E. faecalis* is frequently identified in failed root canal treatment with persistent apical periodontitis (i.e. chronic inflammation and destruction of the hard and soft tissues surrounding the root/s of a tooth caused by etiological agents of endodontic infection) [24]. Persistent apical periodontitis occurs when root canal treatment of apical periodontitis has not adequately eliminated infection within the root canal space. Although interactions between *E. faecalis* and macrophages have been investigated from different perspectives, information is lacking on the effect of prolonged *E. faecalis* infection on differentiating macrophages.

In the present study, *E. faecalis* were identified within the cytoplasm of macrophages that were infected prior to their differentiation, as well as in fully-differentiated macrophages infected by *E. faecalis*. These findings indicate that *E. faecalis* uptake by macrophages is independent of the stage of cellular differentiation. Previous investigations that utilized TEM to examine macrophages showed similar bacteria internalization [15].

Apparently, macrophage differentiation in terms of typical phenotypic markers was not affected by *E. faecalis*. Previous studies using different models of infection have reported variable results [9,16]. In the present study, *E. faecalis* was applied to BMSCs prior to their differentiation and maintained for 8 days. The objective of this “prolonged infection’ model was to mimic clinical conditions where monocytes migrate into a site of chronic infection. Conversely, fully-differentiated macrophages were exposed to *E. faecalis* for a short time only (i.e. 24 h), similar to the protocol used in other studies [16].

The apoptosis assay employed showed that *E. faecalis* infection of BMSCs prior to their differentiation into macrophages significantly reduced the apoptotic activity of the subsequently differentiated macrophages. Such a phenomenon has been reported as one of the strategies used by bacteria to survive within macrophages [25]. Maintaining the viability of infected macrophages enables bacteria to utilize the cells as vehicles for their translocation to different parts of the body and aids in spreading the infection [26]. Replication of the intravacuolar *E. faecalis* within the macrophages is suggestive of their robust survival potential and their ability to avoid destruction by preventing fusion of their encasing vacuoles with intracellular lysosomes [26,27]. Infection of fully-differentiated macrophages with *E. faecalis* for 24 h did not show the same effect on cellular apoptosis. This suggests potential differences in macrophage behavior according to the stage of differentiation.

Polarization of macrophages into the M1 (classical) or M2 (alternative) phenotype reflects differences in the nature of the environmental milieu, which may be the predominantly inflammatory (M1 state) or the homeostatic (M2 state) [28]. Intracellular bacterial infections can skew macrophages toward the M1 or M2 phenotype to increase their chance of intracellular survival [29]. In the present study, *E. faecalis* infection of macrophages caused polarization toward an M1-like phenotype, with a strong expression of CD38 and weak expression of EGR2. The IRF5 protein, a specific marker for M1 polarization that promotes macrophage differentiation into the inflammatory subtype [21,30–32], was highly expressed by the infected macrophages. Identification of the IRF5 protein is a more definitive indication of the M1-like macrophage phenotype. This finding is consistent with previous reports that enterococcal infection stimulates macrophages to polarize into the M1 phenotype through activation of the Toll-like receptor/Myeloid differentiation primary response 88/Microtubule associated protein kinase/Nuclear factor-kappa beta signaling pathways [22]. Macrophage polarization into the pro-inflammatory M1 phenotype has been reported in bacterial infections involving *Listeria monocytogenes, Salmonella typhi, Salmonella typhimurium, Mycobacterium tuberculosis*, salmonella and chlamydial infections [33–35]. Macrophages usually demonstrate plasticity between M1 and M2 subsets, where both phenotypes can transform into each other under different stimulations. Transformation of pro-inflammatory M1 macrophages to an anti-inflammatory M2 phenotype is required for inflammation resolution and healing [36]. In the present study, addition of M2 growth factors to the pre-M0 + *E. faecalis* (i.e., pre-M0 + *E. faecalis* + IL-4 and IL-13) did not affect macrophage polarization, which is suggestive of the inhibition of macrophage plasticity. This feature has been seen previously in diabetic mice, in which deterioration in macrophage function resulted in the cells remaining in the M1 polarization state [37].

Polarization toward the M1 phenotype by human and murine macrophages in response to infection is considered a critical protective mechanism. This is associated with the upregulation of pro-inflammatory cytokines to activate the adaptive immune response [29]. A distinctive cytokine expression profile was identified in the present study when *E. faecalis* infection of the macrophages was initiated prior to their differentiation. Although no noticeable changes were found in the TGF-β1 and TNF-α expression in relation to the control groups, atypical reduction in the pro-inflammatory cytokines IL-1β and IL-12 and the significantly higher expression of IL-10 may explain why *E. faecalis* could alter macrophage polarization into an aberrant M1-like phenotype. This altered functional phenotype may represent a previously-unreported intermediate state within the macrophage phenotypic spectrum, with an inclination toward the M1 side of the spectrum (i.e. M1-like macrophages) [28,38,39]. Interleukin-1β has been shown to have a protective role against microbial infections. This cytokine protects the host against invading pathogens through a cascade of events such as rapid recruitment of neutrophils to the site of infection, activation of endothelial adhesion molecules, stimulation of cytokine production and activation of adaptive immunity [40]. Inhibition of IL-1β production enhances the susceptibility of the host to bacterial infection, a mechanism utilized by some bacteria to overcome the host’s immune response [41]. Interleukin-12 a pro-inflammatory cytokine that links the innate and adaptive immune systems, induces the differentiation of Th-1 cells and activates natural killer cells, thereby protecting the host against infection [42]. Previous studies performed on IL-12 knockout mice reported an increase in the susceptibility of the mice to infection by intracellular pathogens that was caused by defective immune responses [43,44].

Unlike the two aforementioned cytokines, IL-10 is an immunosuppressive cytokine that downregulates the inflammatory immune response and protects the host tissue against the harmful effects of inflammation. However, over-production of this cytokine favors bacterial persistence due to the lack of clearance of the invading pathogens [45]. Production of IL-10 is more destructive to the host in case of intracellular bacterial infections [46]. These results provide insight into the persistence of *E. faecalis* infection and the resistance to inflammation resolution in *E. faecalis*-induced medical and dental conditions, including apical periodontitis [47]. Accordingly, the null hypothesis that ‘prolonged *E. faecalis* infection of macrophage precursors have no effect on the phenotype of the differentiated macrophages and their cytokine expression profile’ has to be rejected.

A realistic limitation of the present study is the divergence that exists between human and murine systems. Because of this diversity, the ideal way to understand human diseases is to perform research on human subjects or human cells. However, from a technical and an ethical point of view, mice may be considered excellent models to study diseases related to humans. Previous studies reported that mice and humans share a significantly high percentage of gene expressions; they also exhibit similarities in many physiological and pathological features [48,49]. This was the basis for the present study to be conducted on murine cells instead of human cells.

## Conclusion

The present study was conducted to detect the effect of *E. faecalis* infection of macrophage precursors on the polarization and inflammatory cytokines profile of the resultant cells. Within the limits of the *in vitro* study, it may be concluded that early exposure to *E. faecalis* infection during macrophage differentiation does not prevent the expression of prototypic macrophage markers. Indeed, the viability of macrophages is increased, while induction of an aberrant M1 phenotype with an altered cytokine expression is evident. This aberrant phenotype is resistant to lineage-determining growth factors that, under normal circumstances, trigger their development into M2 macrophages. The plasticity of macrophages is restricted after infection of the macrophage precursors by *E. faecalis*.

## Supplementary Material

Supplemental Material
