# Supplementary material for: Enterococcus faecalis shifts macrophage polarization toward M1-like phenotype with an altered cytokine profile
Source: J Oral Microbiol. 2021 Jan 4;13(1):1868152. doi: 10.1080/20002297.2020.1868152 (PMC7801083; doi:10.1080/20002297.2020.1868152)
Supplement: Supplemental Material [file ZJOM_A_1868152_SM9623.zip › Supplementray files/Supplementary Data.docx]

**Supplementary data**

Supplementary video shows the process of bacterial uptake by differentiated macrophages. The cells were infected for 6 h with stained bacteria and visualized with confocal laser scanning microscopy in the bright field using the live video mode.
